# A prospective comparative study between percutaneous cannulated screws and Kirschner wires in treatment of displaced intra-articular calcaneal fractures

**DOI:** 10.1007/s00264-022-05521-y

**Published:** 2022-08-12

**Authors:** Hossam El-Azab, Khalaf Fathy Elsayed Ahmed, Abdelrahman Hafez Khalefa, Ashraf Rashad Marzouk

**Affiliations:** Orthopaedics and Traumatology Department, Sohag Faculty of Medicine, Sohag University, Sohag, Egypt

**Keywords:** Percutaneous fixation, Intra-articular calcaneal fractures, Minimally invasive surgery, Cannulated screws, K-wires, Prospective comparative study

## Abstract

**Purpose:**

Several minimally invasive procedures were used to treat displaced intra-articular calcaneal fractures (DIACFs). No agreement among different authors about either the ideal fixation method or which technique is minimally invasive. The aim of this study was to compare functional and radiographic outcomes of two minimally invasive techniques in treatment of Sanders type II and III DIACFs by using K-wires or cannulated screws without bone grafts.

**Methods:**

A prospective randomized controlled study was conducted on 28 patients (34 feet) with Sanders type II or III DIACFs, treated by closed reduction and fixation using cannulated screws or K-wires, at the Orthopedics Department of Sohag University Hospital, between April 2020 and February 2022. Functional assessment was done by American Orthopedic Foot and Ankle Society (AOFAS) score and VAS for pain. Radiographic assessment was done by measurement of three calcaneal angles (Gissane, Böhler’s, and posterior facet inclination angles) and three calcaneal distances (height, length, and width of the calcaneus).

**Results:**

Mean ages of patients at the time of operation were 34.8 years for the cannulated screw group and 36.6 years for the K-wire group. A vast majority of patients were males (78.6%). Involvement of the right side in the cannulated screw group was 57.1% and that in the K-wire group was 47.9%. Mean operative time was significantly shorter among the K-wire group (42 min) compared to the cannulated screw group (57 min). Mean AOFAS score was higher among the cannulated screw group (85.9 points) compared to the K-wire group (75.8 points). Final VAS was significantly better among the cannulated screw group compared to the K-wire group. Mean time of radiographic union in the cannulated screw group was 8.9 weeks and that in the K-wire group was 10.1 weeks.

**Conclusion:**

Both techniques avoided wound complications associated with ORIF with the advantage of a shorter hospital stay. Patients in the cannulated screw group had better functional and radiographic outcomes and a lower rate of subtalar arthritis than patients in the K-wire group. K-wires had advantages of reduced operative time, and easy removal as an outpatient procedure.

## Introduction

The calcaneus is the most commonly fractured tarsal bone; accounting for 60% of tarsal fractures, and 1–2% of all fractures with a majority of fractures occurring in males at age of 21–45 years, thus the socioeconomic implications are striking. About 75% of calcaneal fractures are intra-articular and result from axial loading mechanisms due to falling from a height or motor car accidents [1].

Treatment of displaced intra-articular calcaneal fractures (DIACFs) remains controversial and challenging due to its complex anatomy, complex articulations, delicate soft tissue coverage, and associated complications. Current management options for DIACFs are divided into four categories: conservative treatment, open reduction and internal fixation (ORIF), minimally invasive surgery (MIS), and ORIF with primary subtalar arthrodesis [2]. ORIF is associated with a wound complication rate of 16 to 25% including wound infection, dehiscence, and necrosis [2]. Conservative treatment may not be acceptable in all patients; while it may be preferred in patients with severe comminution, elderly patients, and patients with medical comorbidities in which surgical management is contraindicated, it may not be acceptable in young, active patients with DIACFs [3].

MIS for DIACFs strives to strike a balance between ORIF and conservative treatment. MIS techniques have a decreased rate of wound complications but carry the risk of inadequate reduction [4]. Several MIS techniques have been used to treat DIACFS including arthroscopic-assisted reduction and fixation [5], Ilizarov device [6], interlocking calcaneal nails [7], sinus tarsi approach and percutaneous plate [8], and percutaneous screws or K-wires.

Currently, there is neither agreement in the techniques described by surgeons to meet the criteria of being minimally invasive nor consensus among different authors regarding the ideal fixation method. There is no prospective study that compared percutaneous K-wires with percutaneous cannulated screws in treatment of DIACFs.

The current prospective randomized controlled study aimed to compare the functional and radiographic outcomes of two minimally invasive techniques in treatment of Sanders type II and III DIACFs using K-wires or cannulated screws without bone grafts.

## Materials and methods

### Materials

The current prospective randomized controlled comparative study was conducted on 28 patients (34 feet) with Sanders type II and III DIACFs (6 patients with bilateral fractures and 22 patients with unilateral fracture), at Orthopedics Department of Sohag University Hospital, between April 2020 and February 2022. The patients of the study were randomly operated on and divided into two groups (cannulated screw and K-wire groups); each group was composed of 11 unilateral and three bilateral fractures. The method of randomization done was the stratified randomization one [9].

The inclusion criteria were Sanders type II or III closed DIACFs in adult patients of duration ≤ 14 days. The exclusion criteria were open fractures, old fracture (> 14 days), non-displaced fractures, extra-articular fractures, fractures in children, or previously fractured calcaneus.

At first presentation to the emergency unit, a detailed history was taken and general examination following the advanced trauma life support (ATLS) protocol for detection of any associated injuries or fractures was done for all patients. Local examination of the injured foot for the side of injury, swelling, and neurovascular condition was done.

Informed written consent with risk explanation was obtained from all patients. The study was approved by Scientific and Ethical Committees of Sohag Faculty of Medicine.

### Methods

#### Radiographic evaluation

**Fig. 1:**
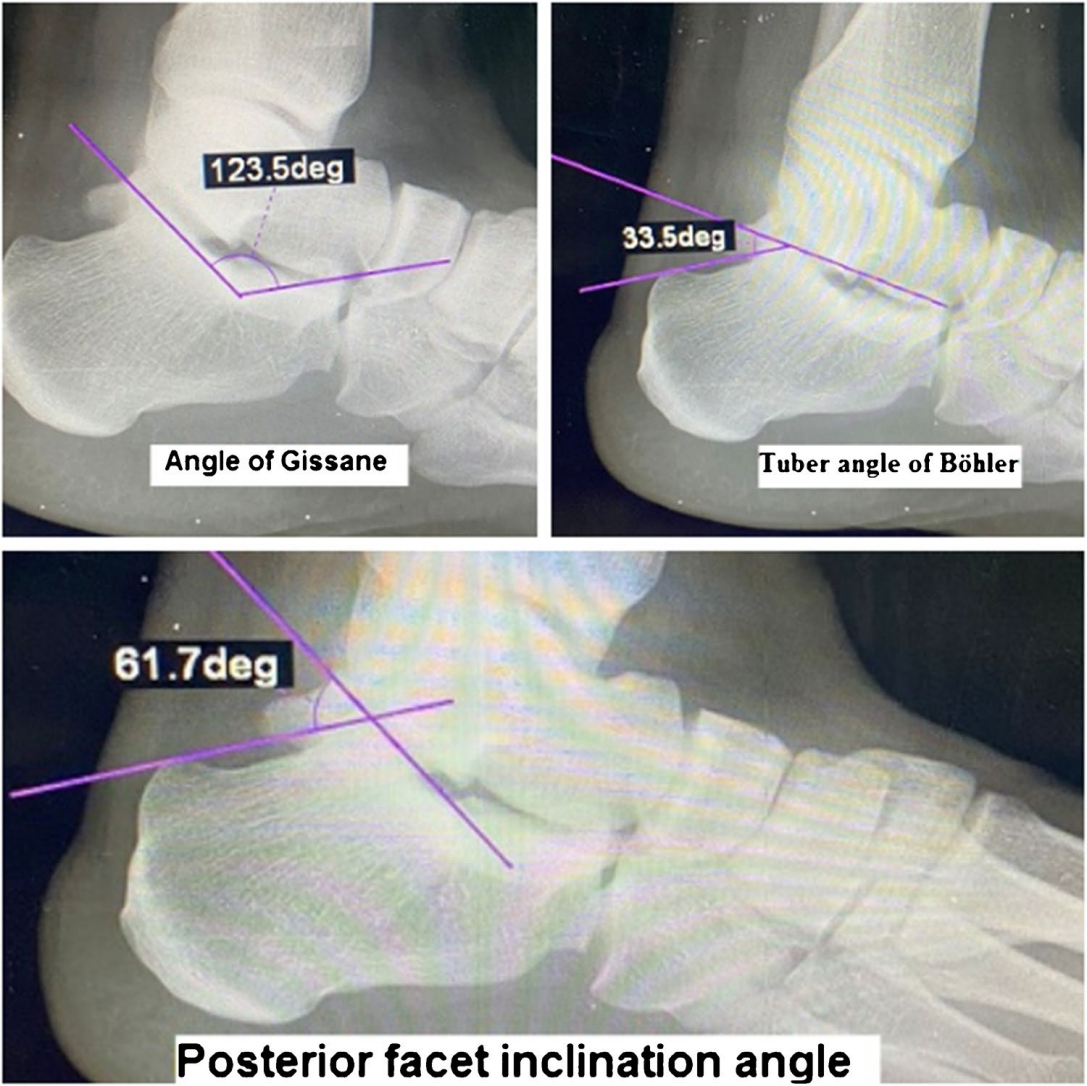
Three calcaneal angles used in evaluation of calcaneal fractures

**Fig. 2:**
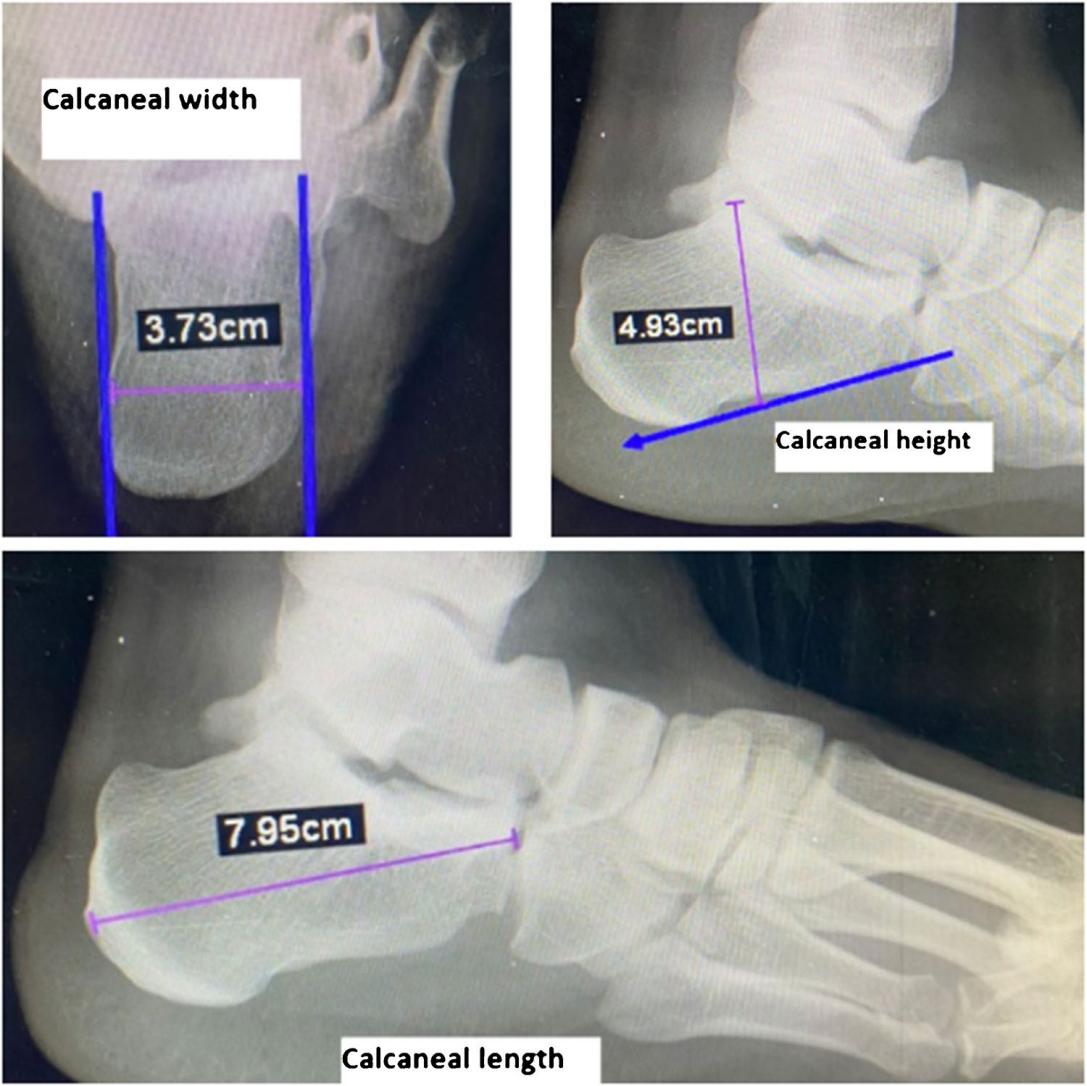
Three calcaneal distances used in evaluation of calcaneal fractures

A. *Plain radiography*: Lateral hindfoot and axial calcaneal views with radiographic interpretation of three calcaneal angles and three calcaneal distances.
  The three calcaneal angles measured were as follows: (a) Tuber angle of Böhler: formed at the intersection of a line connecting the highest point of calcaneal tuberosity to the highest point of posterior facet with a line connecting the highest point of posterior facet to the highest point of anterior calcaneal process [10]. It is normally 20°–40°. (b) Crucial angle of Gissane: formed between the line of posterior calcaneal facet and the line from the sulcus calcanei to the tip of anterior calcaneal process. It is normally 120°–145° [10]. (c) Posterior facet inclination (Sarrafian) angle: formed by the two intersecting lines drawn along the surface of the posterior facet and the upper surface of the calcaneal tuberosity. It is normally 55°–75° [10] (Fig. 1).
  The three calcaneal distances measured (mm) were as follows: (a) Calcaneal height: a line perpendicular on the calcaneal axis to the highest point of the posterior facet in lateral view. (b) Calcaneal length: measured from the most posterior point of the tuberosity to the center of the calcaneocuboid joint in lateral view. (C) Calcaneal width: the length of a perpendicular line connecting two parallel lines drawn tangent to the widest part of calcaneal tuberosity in axial view [10] (Fig. 2).
B. *Computed tomography (CT) scan*: Computed tomography scan of the fractured calcaneus in axial, coronal, and sagittal planes.

#### Classification of fractures

Sanders [11] and Essex-Lopresti [12] classification systems were used for all fractures.

#### Operative technique

**Fig. 3:**
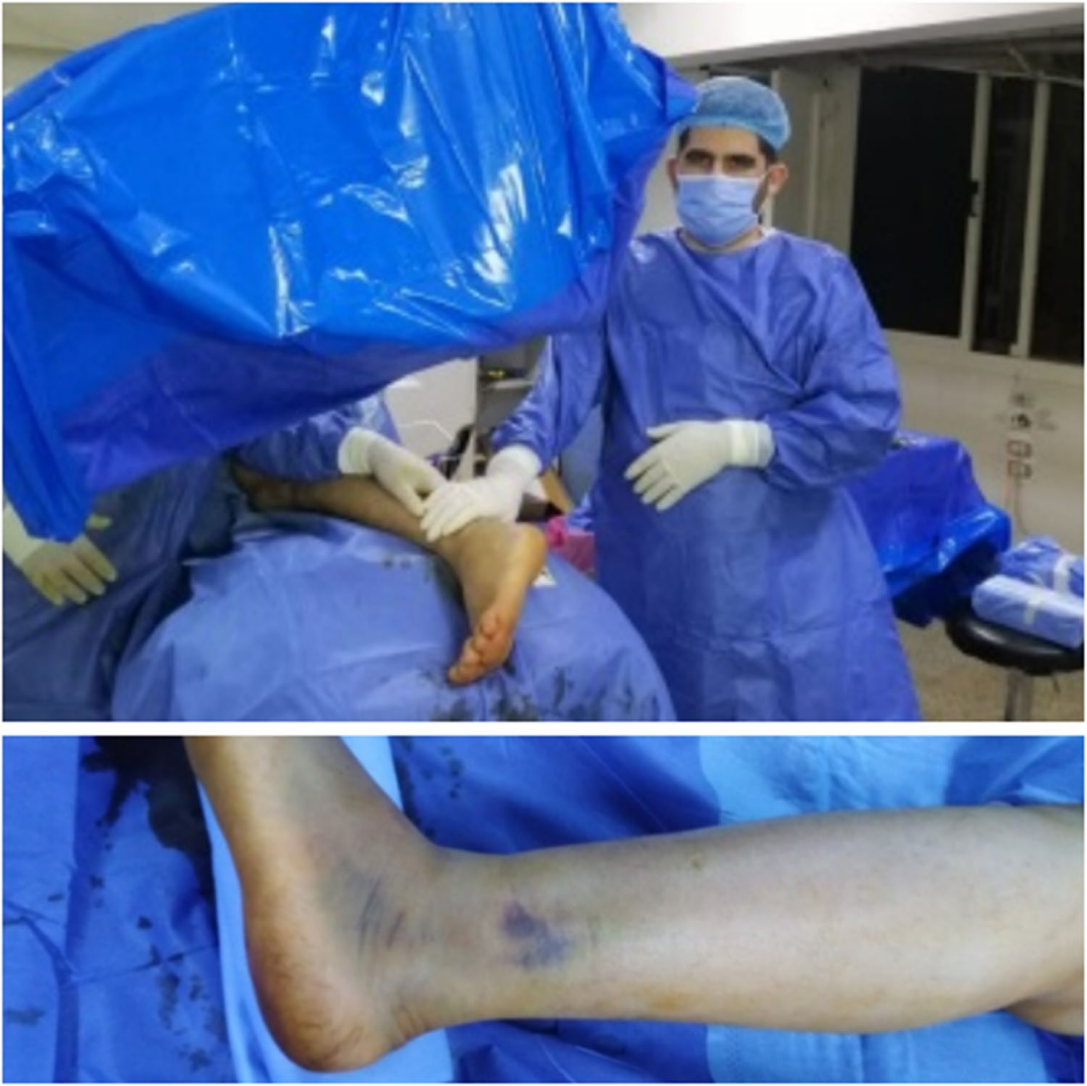
C-arm entered the operative field opposite the side where the surgeon was standing. Patient positioning with foot elevated over closed gown pack

Patients were operated on within the first two weeks of trauma. The presence of a wrinkle sign was not considered in determination of surgical timing as we used the percutaneous approach. The patient was positioned in a lateral decubitus position with the affected side up on the radiolucent operating table. The affected leg was positioned flat on a firm bump to support a perfect lateral position of the foot with or without the use of a tourniquet. Operations were done under spinal anaesthesia with C-arm guidance, and the surgeon stood on the opposite side of C-arm entry (Fig. 3).

## Reduction of fractures

**Fig. 4:**
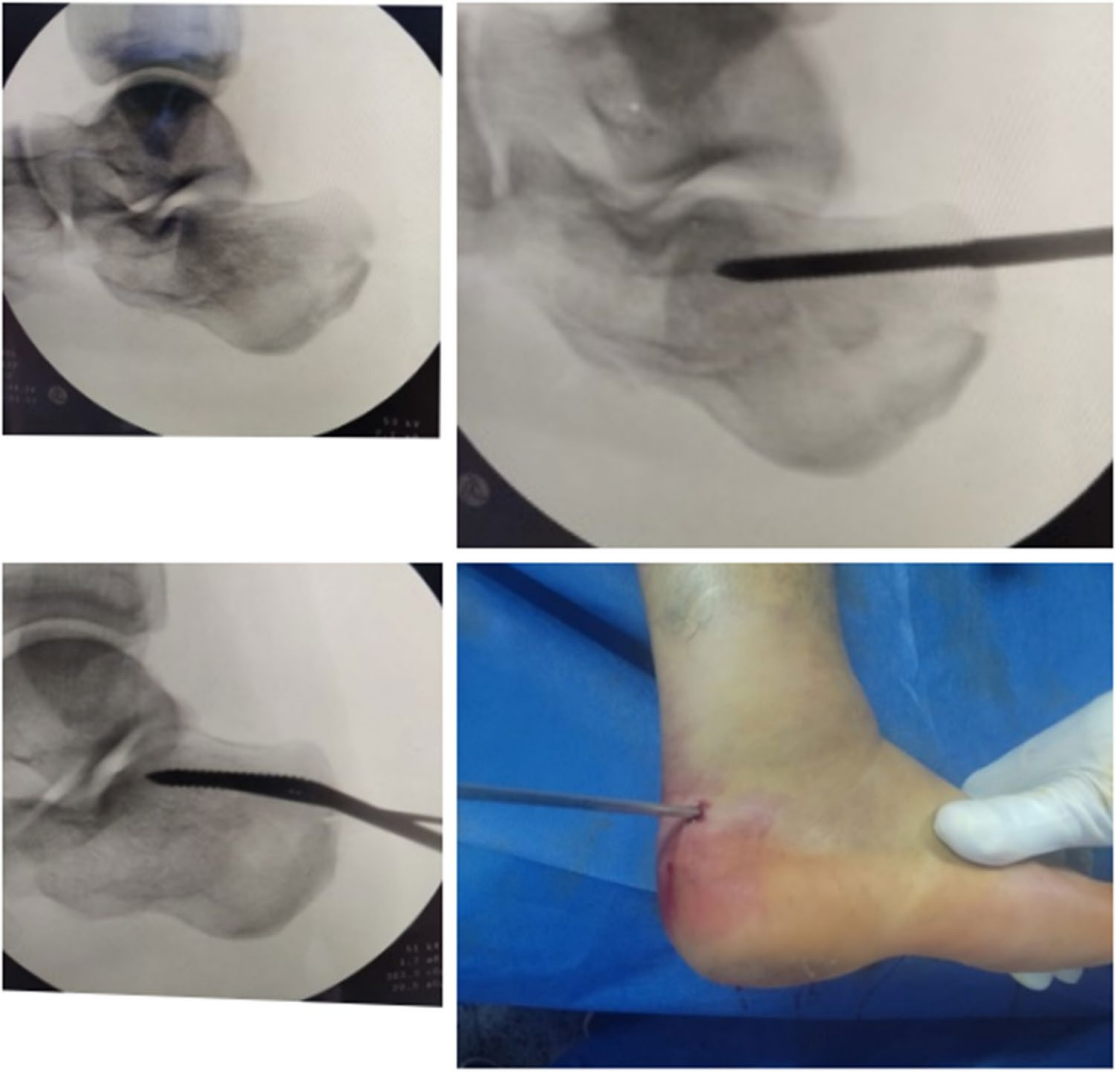
Reduction of tongue-type fractures

1. Percutaneous reduction techniques for tongue-type fractures
  A 3- to 4-mm Schanz screw was inserted into the facet (tongue) fragment from postero-superior lateral to the Achilles tendon directed towards the antero-inferior margin of the posterior facet fragment in line with deformity. The dorsum of the foot was held with the palm of one hand and the Schanz screw with the other hand. Both thumbs were placed on the plantar surface of the foot under the middle of the calcaneus. Reduction was performed in three steps: first, the fracture fragments were disengaged by exaggerating the varus/lateral angulation of fracture; second, the Schanz screw was used as a joystick to achieve reduction, and the midfoot and Schanz screw were forced plantarly (by employing downward pressure like breaking a stick) using the thumbs as a fulcrum, so the fragment was pushed firmly against the corresponding articular surface of the talus; finally, the foot was forced into the valgus to bring the posterior facet adjacent to the sustentaculum tali. The Schanz screw used for reduction was usually bent to an angle greater than 30° (Fig. 4).
2. Percutaneous reduction techniques for joint-depression fractures

**Fig. 5:**
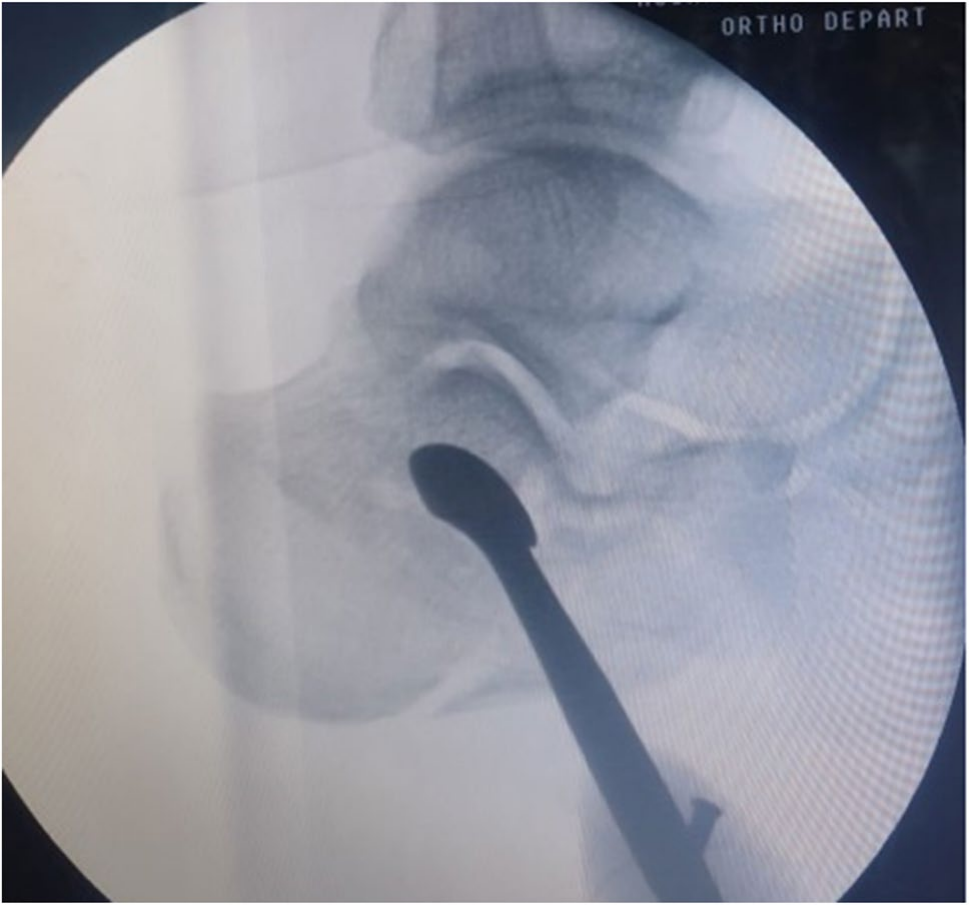
Elevation of the posterior facet using periosteal elevator

Reduction of calcaneal tuberosity was the first step in the technique. The depressed facet would not reduce until calcaneal tuberosity was moved out of an obstructing position. Manipulation of calcaneal tuberosity fragment using a 6.5-mm Schanz screw inserted from lateral to medial in calcaneal tuberosity either perpendicular to the length of calcaneus or at an angle from posterior lateral to medial side anteriorly. The starting point was posterior in the tuberosity and midway from top to bottom. The Schanz screw was used to do distraction by axial traction while counter-traction is provided by an assistant at the forefoot level with the ankle held in plantar flexion. The force was directed laterally for correction of varus angulation of the tuberosity if needed to be corrected. Manipulation was done for restoration of length and height of the calcaneus and achievement of correction of the inferior and medial translation. Apply manual traction on laparotomy gauze on both sides of the Schanz screw. Assessment of reduction of posterior facet fragment or fragments on lateral hindfoot and axial (calcaneal) views, if not reduced, a 1–2-cm small lateral incision was done distal and anterior to the tip of lateral malleolus at the sinus tarsi; then, a periosteal elevator or curved artery forceps was inserted through the incision to elevate up the depressed posterior facet fragments, and to achieve anatomical reduction (Fig. 5).

## Percutaneous fixation techniques

**Fig. 6:**
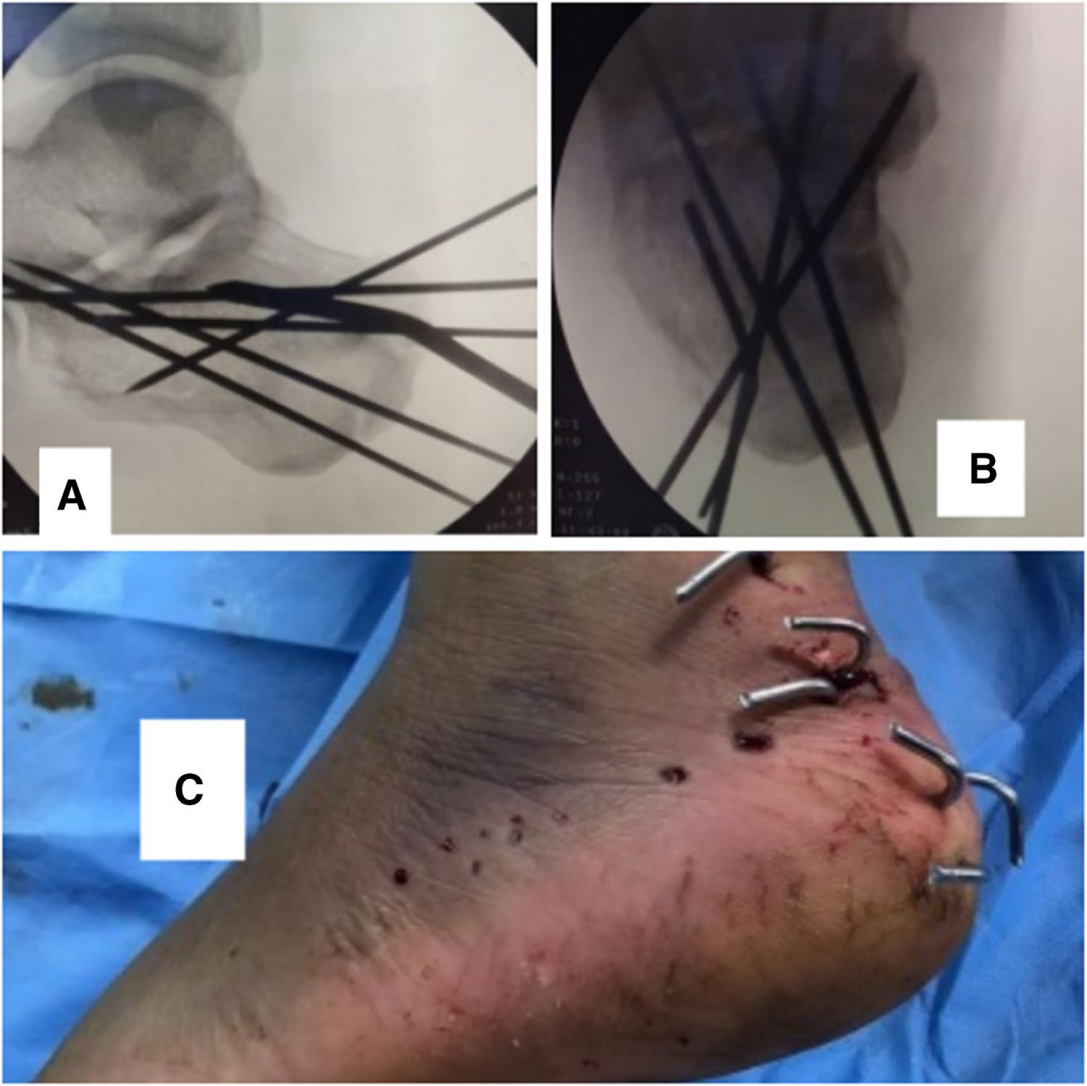
Appropriate length and position of K-wires were confirmed by using the C-arm: lateral (**A**) and axial (**B**) views. K-wires were cut and bent outside the skin (**C**)

1. K-wire fixation
  Multiple K-wires of 1.6- or 1.8-mm K-wires were inserted. A number of K-wires depended on fracture type and degree of comminution. K-wires secured both the reduced fragments and the overall calcaneal alignment. Two K-wires were inserted from the posterolateral aspect and directed medially to the sustentaculum tali. Two K-wires were inserted on each side of tendon Achilles insertion directed subarticularly to anterior process of the calcaneus. Additional K-wires may be inserted if needed. Appropriate length and position of K-wires were confirmed by using the C-arm (Fig. 6a and b), and then K-wires were cut and bent outside the skin (Fig. 6c).
2. Cannulated screw fixation

**Fig. 7:**
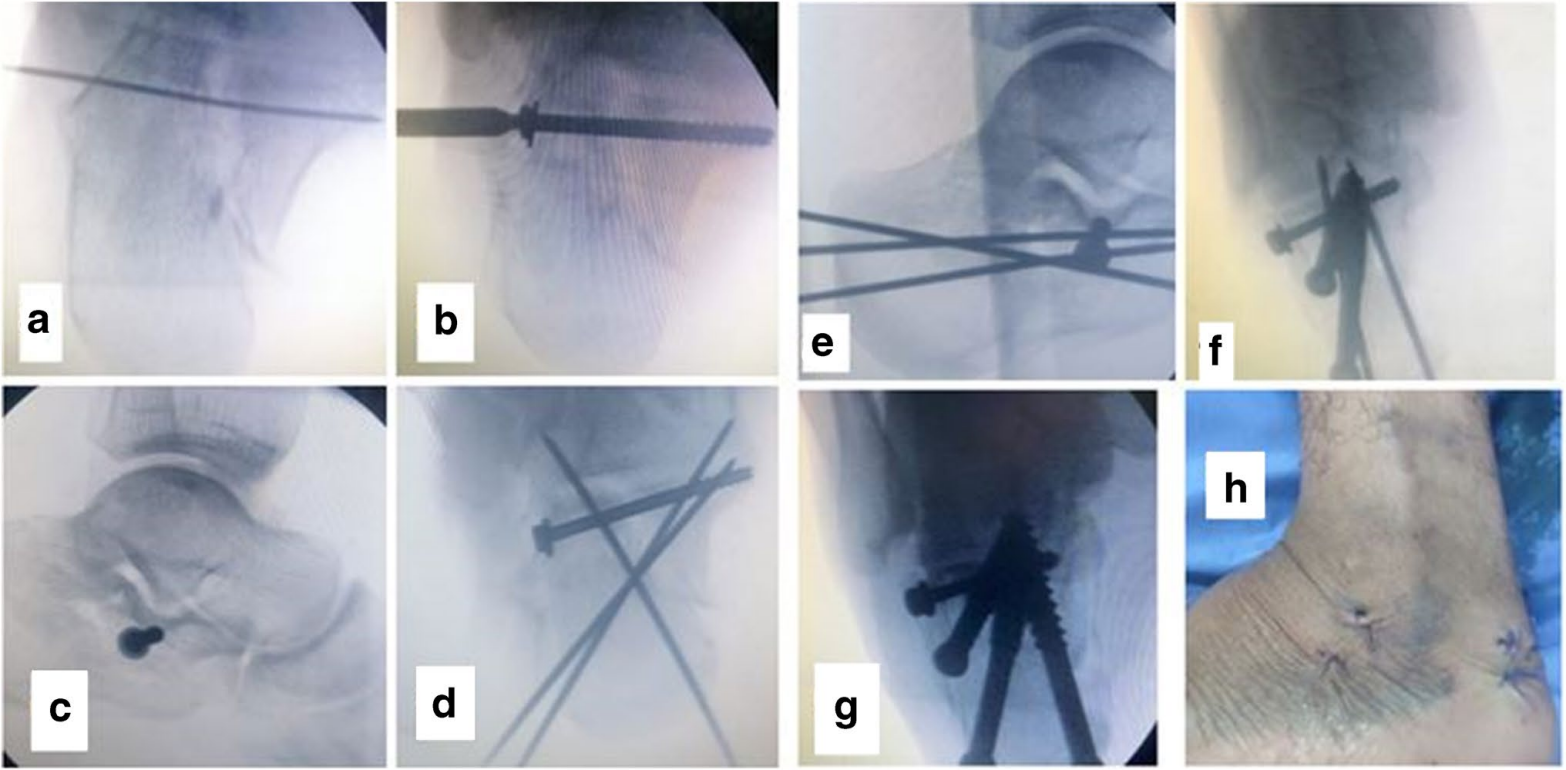
Percutaneous cannulated screw insertion sequence and confirmation of position by lateral and axial views (**a**–**g**), and clinical photograph of skin closure (**h**)

Temporary fixation of the depressed posterior facet fragment which elevated up was done using an appropriate guide wire for a cannulated screw that was inserted from lateral to medial side at subarticular level directed to the sustentaculum tali (Fig. 7a). Cannulated drill bit was used to drill a hole over the guide wire. Fixation was done using a 4.0-mm partially threaded cannulated screw inserted over the guide wire with a washer (to improve fixation); good screw purchase on the medial side was ensured. Axial view was used to assess appropriate position of the guide wire and screw length. The length of this screw was about 35 to 45 mm (Fig. 7b and c).

Temporary fixation of fracture by using percutaneous guide wires for cannulated screw for fixation of tuberosity fragment to the sustentaculum tali and to anterior process of calcaneus (Fig. 7d and e). Determine the length of screws on lateral view, and then the cannulated drill bit was used to drill a hole at the screw entry only (to ensure good purchase of screws in cancellous bone) over the guide wires. Fixation of fracture fragments was done using partially threaded cannulated rafting screws 6.5 or 7.3 mm (length of threads was 32 mm) with washers, thus maintaining the length and posterior valgus angulation of calcaneus (Fig. 7f and g). Screws were placed flushed with the back of tuberosity to avoid prominence of screw heads. Additional screws may be inserted to compress widening of the calcaneus at the tuberosity level. The final screw lengths and positions were confirmed with lateral and axial views.

## Reduction of calcaneal widening and evacuation of haematoma

By compressing the heel between two hands after articular fragments have been reduced, and after permanent fixation of fractures.

## Skin closure and post-operative care

Stab wound and sites of screw insertion were closed with sutures (Fig. 7h). Below knee slab or cast was applied with leg elevation and active movement of the toes and ankle. IV antibiotics were given to all patients for three days then completed oral for two weeks. Post-operative non-weight bearing with crutches mobilization for six to 12 weeks according to each case.

## Follow-up evaluation

**Fig. 8:**
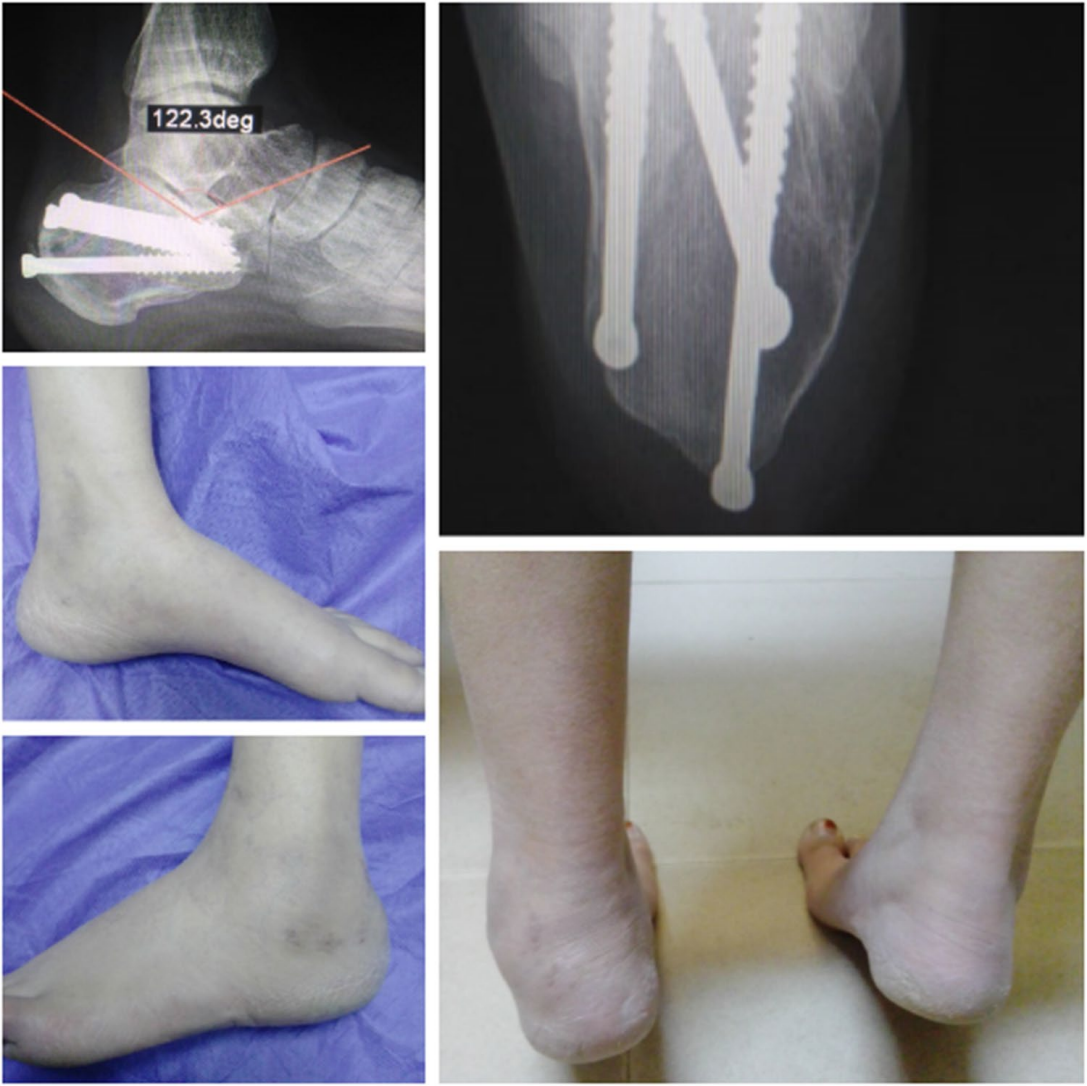
Final radiographic and clinical follow-up of patient with cannulated screw fixation

**Fig. 9:**
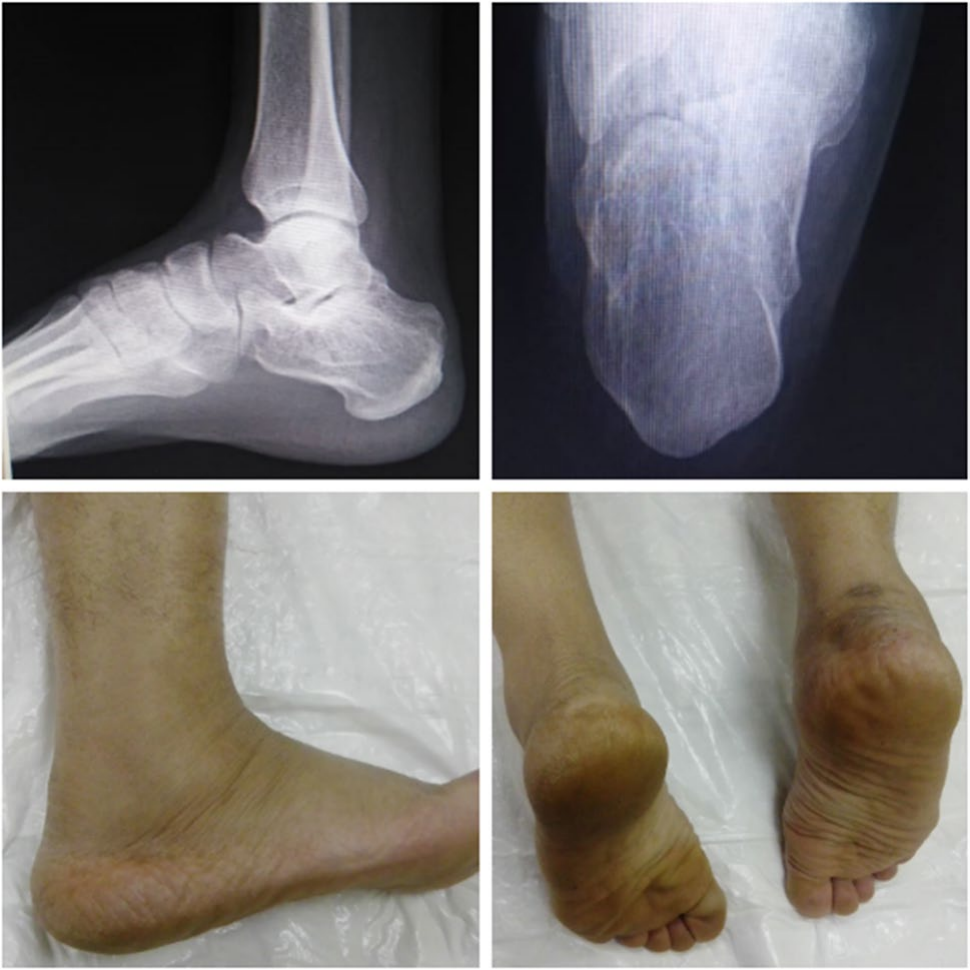
Final radiographic and clinical follow-up of patient with K-wire fixation

Follow-up at outpatient clinic of Sohag University Hospitals at scheduled intervals: two weeks, six weeks, 12 weeks, six months, and at 12 months (Figs. 8 and 9).

## Clinical evaluation

**Fig. 10:**
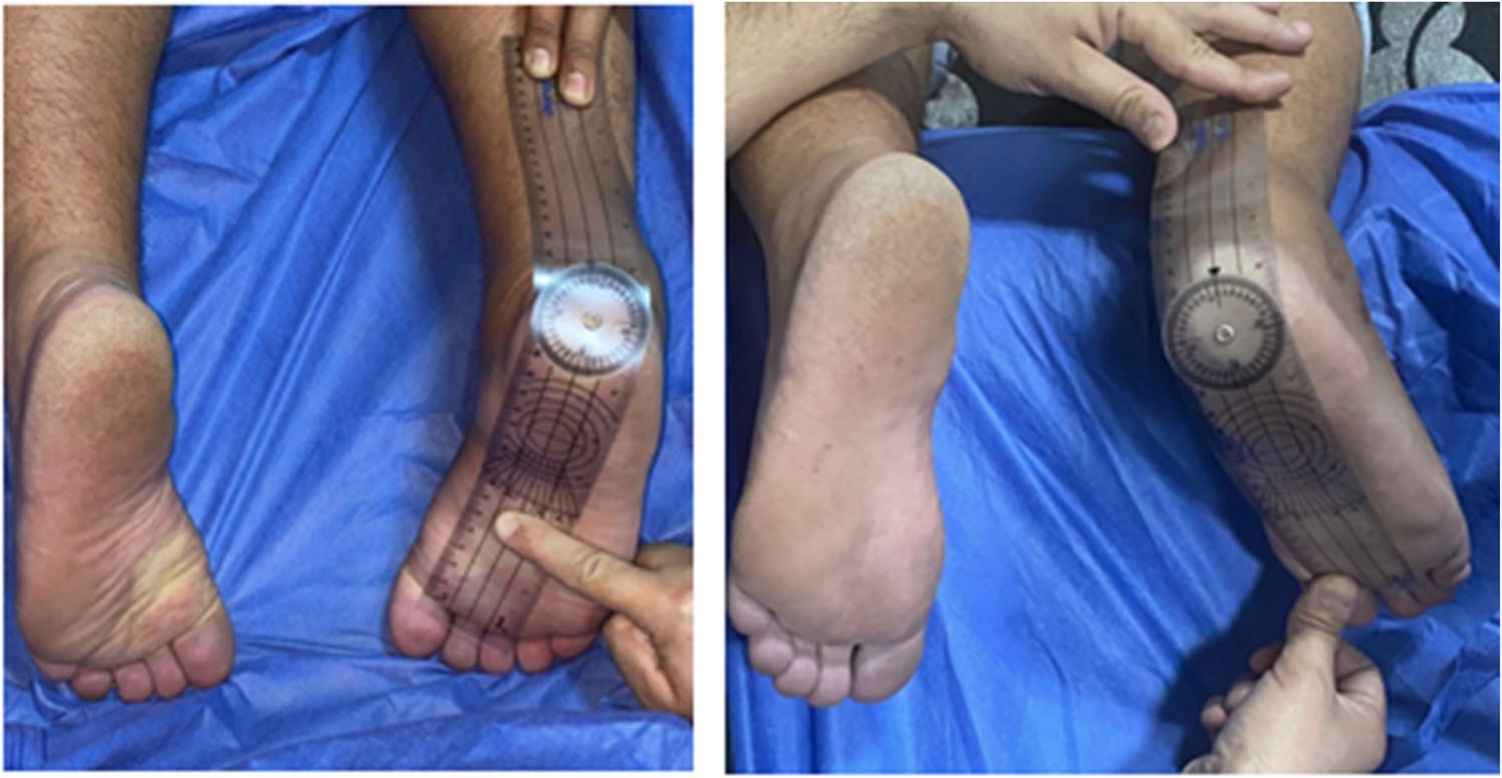
Goniometric measurements of subtalar joint inversion and eversion

Two weeks post-operatively, sutures were removed. Six weeks post-operatively, K-wires were removed. Partial and full weight-bearing were allowed according to the condition. Physical assessment by the American Orthopedic Foot & Ankle Society (AOFAS) ankle-hindfoot scoring system [13] at months post-operatively. Patients were assessed for pain by 11 points (0–10) Visual Analogue Scale (VAS) [14]. Measurements of subtalar ROM (inversion and eversion) were done by a goniometer using a technique of Kimberly [15] (Fig. 10).

## Radiographic evaluation

Patients were evaluated for adequacy of reduction of subtalar joint, stability of fixation, fracture union, restoration of normal anatomy, and measurement of radiographic parameters as done pre-operatively. Subtalar arthritis evaluation by the Paley grading system (PGS) [16].

### Statistical analysis

Statistical Package for Social Sciences (IBM-SPSS), version 25 (IBM Corporation, Chicago, USA; August 2017), was used for statistical data analysis. Data were expressed as mean, standard deviation (SD), number, and percentage. Mean and standard deviation were used as descriptive values for quantitative data. Student *t*-test was used to compare the means between two groups. Mann–Whitney test was used instead of Student *t*-test for non-parametric data to compare medians rather than means. Pearson chi-square test was used to compare percentages of qualitative variables, and Fisher’s exact test was used instead for non-parametric data. Paired *t*-test was used to compare means of the same variable at different periods of time, and Wilcoxon test was used for non-parametric data. For all these tests, the level of significance (*P*-value) was explained as follows: no significance (*P* > 0.05), significance (*P* < 0.05), and high significance (*P* < 0.001).

## Results

The mean age of patients at the time of operation was 34.8 ± 9.3 (range, 22–55) years for the cannulated screw group and 36.6 ± 12 (range, 23–57) years for the K-wire group. The vast majority of patients were males (78.6%). Male predominance was more evident among the patients of the cannulated screw group. Involvement of the right side in the cannulated screw group was 57.2% and that in the K-wire group was 47.9%. The mean operative time was significantly shorter among the K-wire group at 42 (range, 35–50) min compared to the cannulated screw group at 57 (range, 45–65) min. The mean follow-up period was 12.4 ± 3.7 (range, 8–18) months in the cannulated screw group, and 12.1 ± 3.9 (range, 8–19) months in the K-wire group. There was a slight rise of smokers among the cannulated screw group. There was no significant difference among two groups regarding mechanism of injury, Sanders and Essex-Lopresti classifications, time lapsed to surgery, hospital stays, or place of trauma (Table 1).

A vast majority of patients had isolated DIACFs. Two (14.3%) patients in the cannulated screw group had associated injuries; one had wedge L1 which was managed by a brace, and the other had burst L4 and distal radius fractures which were managed operatively. Six (42.9%) patients in the K-wire group had associated injuries; two of them had wedge L1 which were managed conservatively, two of them had burst D12 which were managed operatively, one had distal radius fracture which was managed conservatively, and one had distal radius and scaphoid fractures which were managed operatively.

**Table 1:** Demographics data of the two study groups

Characteristics		Cannulated screw group	K-wire group	*P*-value
Total patients:feet		14:17	14:17	
Age at time of operation (years)				
Mean ± SD (range)		34.79 ± 9.26 (22–55)	36.57 ± 12 (23–57)	0.3315 (NS)
Sex				
Males, no. (%)		13 (92.9%)	9 (64.3%)	0.165 (NS)
Females, no. (%)		1 (7.1%)	5 (35.7%)	
Side affected				
Unilateral, no. (%)	Right	8 (57.2%)	6 (42.9%)	0.673 (NS)
	Left	3 (21.4%)	5 (35.7%)	
Bilateral, no. (%)		3 (21.4%)	3 (21.4%)	
Mechanism of injury				
Falling from height, no. (%)		12 (85.7%)	11 (78.6%)	0.828 (NS)
Falling down stairs, no. (%)		1 (7.1%)	1 (7.1%)	
Road traffic accident, no. (%)		1 (7.1%)	2 (14.3%)	
Sanders classification				
Type II		5 (29.4%)	5 (29.4%)	1.00 (NS)
Type III		12 (70.6%)	12 (70.6%)	
Essex-Lopresti classification				
Joint depression type		12 (70.6%)	13 (76.5%)	1.00 (NS)
Tongue type		5 (29.4%)	4 (23.5%)	
Smoking				
Non-smokers		8 (57.1%)	10 (71.4%)	0.430 (NS)
Smokers		6 (42.9%)	4 (28.6%)	
Time lapsed from trauma to surgery (days)				
Mean ± SD (range)		6 ± 4.54 (1–14)	5.07 ± 4.39 (1–14)	0.586 (NS)
Operative time (minutes)				
Mean ± SD (range)		57.06 ± 5.88 (45–65)	42.06 ± 5.61 (35–50)	< 0.001 (HS)
Hospital stays (days)				
Mean ± SD (range)		1.64 ± 0.50 (1–2)	1.71 ± 0.61 (1–3)	0.737 (NS)
Follow-up period (months)				
Mean ± SD (range)		12.36 ± 3.69 (8–18)	12.14 ± 3.86 (8–19)	0.4409 (NS)
Place of trauma				
Home, no. (%)		9 (64.3%)	10 (71.4%)	0.591 (NS)
Work, no. (%)		4 (28.6%)	2 (14.3%)	
Street, no. (%)		1 (7.1%)	2 (14.3%)	

### Functional outcomes

The mean AOFAS score at the final follow-up was higher among the cannulated screw group (85.9 ± 8.3 (range, 70–100) points) compared to that in the K-wire group (75.8 ± 9.7 (range, 60–90) points). There were more satisfactory (excellent and good) outcomes among the cannulated screw group (82.3%) compared to those in the K-wire group (58.8%). The mean VAS score showed non-significant differences between the two groups both pre-operatively and at fourth week post-operatively. However, the final VAS was significantly better among the cannulated screw group (1 ± 0.8) compared to the K-wire group (1.7 ± 0.9). The mean subtalar ROM (inversion and eversion) was significantly higher among cannulated screws at 25 ± 5.3 (range, 15–35) compared to the K-wire group at 21.2 ± 5.2 (range, 15–30) degrees. Longer duration was needed for patients until full weight-bearing in the K-wire group at 14.4 ± 1.8 (range, 10–16) weeks compared to the cannulated screw group at 12 ± 1.1 (range, 10–14) weeks. The mean time needed for patients to return to their works was significantly longer among the K-wire group at 5.4 ± 1 (range, 3–7) months compared to that among the cannulated screw group at 4.3 ± 0.8 (range, 3–6) months (Table 2).

**Table 2:** Functional outcomes of the two study groups

Outcomes	Cannulated screw group	K-wire group	*P*-value
AOFAS ankle-hindfoot score at final follow-up (points)			
Mean ± SD (range)	85.9 ± 8.3 (70–100)	75.8 ± 9.7 (60–90)	0.0014 (S)
Excellent, no. (%)	10 (58.82%)	2 (11.76%)	0.016 (S)
Good, no. (%)	4 (23.53%)	8 (47.06%)	
Fair, no. (%)	3 (17.65%)	7 (41.18%)	
Poor, no. (%)	0 (0.0%)	0 (0.0%)	
VAS score for pain (points), mean ± SD (range)			
Pre-op	6.8 ± 0.9 (5–8)	7.3 ± 0.5 (7–8)	0.06 (NS)
4th weeks post-op	3.3 ± 1.3 (2–6)	3.5 ± 0.9 (2–5)	0.463 (NS)
Final follow-up	1 ± 0.8 (0–3)	1.7 ± 0.9 (1–3)	0.050 (S)
Subtalar ROM (eversion and inversion) (°)			
Mean ± SD (range)	25 ± 5.3 (15–35)	21.2 ± 5.2 (15–30)	0.02 (S)
Time of full weight-bearing (weeks)			
Mean ± SD (range)	12 ± 1.1 (10–14)	14.4 ± 1.8 (10–16)	0.0001 (HS)
Time of return to work (months)			
Mean ± SD (range)	4.3 ± 0.8 (3–6)	5.4 ± 1 (3–7)	0.003 (S)
Complications			
Superficial infections	0 (0%)	3 (17.65%)	
Deep infections	0 (0%)	0 (0%)	
Peroneal tendon subluxation	1 (5.88%)	0 (0%)	
Prominent hardware	1 (5.88%)	0 (0%)	
Subtalar joint arthritis (PGS)			
Grade 0	11 (64.7%)	8 (47%)	
Grade 1	4 (23.5%)	7 (41.2%)	
Grade 2	2 (11.8%)	2 (11.8%)	
Grade 3	0 (0%)	0 (0%)	

### Complications

Subtalar arthritis was evaluated according to PGS. In the cannulated screw group, grade 1 is four (23.5%) feet and grade 2 is two (11.8%) feet. In the K-wire group, grade 1 is seven (41.2%) feet and grade 2 is two (11.8%) feet, with no grade 3 in both groups. In the K-wire group, three feet (17.65%) had superficial pin track infection, with no deep infections reported in both groups. In the cannulated screw group, one foot (5.9%) developed peroneal subluxation and tendinitis which was managed conservatively, and one foot (5.9%) developed prominent screws which was managed by removal after solid union (Table 2).

### Radiographic outcomes

The mean time of radiographic solid union was 8.9 ± 1.6 (range, 6–12) weeks in the cannulated screw group and 10.1 ± 1.5 (range, 8–12) weeks in the K-wire group. The range of screws used was two to four screws, and the range of K-wires used was two to six K-wires. Regarding the angle of Gissane, there was no significant difference between two groups pre-operatively; however, both of post-operative and final follow-up measurements were lower among the K-wire group compared to those among the cannulated screw group. Regarding the Böhler’s and posterior facet inclination angles, no significant differences were found between the two groups pre-operatively, post-operatively, or at the final follow-up. Regarding the measurements of three calcaneal distances, the pre-operative, post-operative, and final follow-up measurements of the calcaneal height, length, and width were lower among the K-wire group compared to those among the cannulated screw group with significant differences except for both post-operative calcaneal length and final follow-up calcaneal width which showed no significant differences between two groups (Table 3).

**Table 3:** Radiographic outcomes of the two study groups

Outcomes	Cannulated screw group	K-wire group	*P*-value
Time of radiographic solid union (weeks)			
Mean ± SD (range)	8.9 ± 1.6 (6–12)	10.1 ± 1.5 (8–12)	0.016 (S)
Number of screws or K-wires, no. (%)			
Range	2–4	2–6	** < **0.001 (HS)
2	6 (35.3%)	1 (5.88%)	
3	8 (47.05%)	2 (11.76%)	
4	3 (17.65%)	4 (23.53%)	
5	0 (%)	9 (52.95%)	
6	0 (%)	1 (5.88%)	
Angle of Gissane, mean ± SD (range) (°)			
Pre-op	130.2 ± 9.8 (112.4–146.4)	125.9 ± 16.4 (96.1–151.3)	0.366 (NS)
Post-op	129 ± 4.1 (120.7–134.6)	122.2 ± 7.5 (110.6–132.7)	0.003 (S)
Final follow-up	127.9 ± 6.7 (119.6–148)	111.9 ± 13.8 (91.7–140)	0.001 (S)
Böhler’s angle, mean ± SD (range) (°)			
Pre-op	9.7 ± 11.6 (− 10 to 25.6)	9.95 ± 11.27 (–15.4 to 26.3)	0.955 (NS)
Post-op	25.2 ± 7.2 (12–36.3)	27.2 ± 6.7 (8–39.8)	0.434 (NS)
Final follow-up	24.3 ± 7.7 (10–40)	22.2 ± 7.1 (12.4–38.7)	0.453 (NS)
Posterior facet inclination angle, mean ± SD (range) (°)			
Pre-op	48.3 ± 10.6 (23.1–63.6)	44.34 ± 14.29 (24.3–74.8)	0.376 (NS)
Post-op	58.3 ± 9.1 (42.4–74.5)	57 ± 14 (34.1–77)	0.779 (NS)
Final follow-up	58 ± 9.2 (40–73)	51.2 ± 13 (33.1–81.8)	0.104 (NS)
Calcaneal height, mean ± SD (range) (mm)			
Pre-op	44.8 ± 3 (38.7–50)	34.1 ± 3 (31–37.8)	** < **0.001 (HS)
Post-op	49.7 ± 3 (45.7–53)	42.3 ± 5.5 (34.5–47)	0.02 (S)
Final follow-up	49.3 ± 2 (45–53)	41.4 ± 4.6 (33–46)	0.001 (S)
Calcaneal length, mean ± SD (range) (mm)			
Pre-op	85 ± 4.6 (73.6–89.7)	69.7 ± 11 (50–79)	0.019 (S)
Post-op	88.6 ± 3.9 (83.4–95.5)	76.2 ± 12.7 (51–85)	0.064 (NS)
Final follow-up	88.5 ± 3.4 (83–94)	78 ± 10.8 (51–85)	0.001 (S)
Calcaneal width, mean ± SD (range) (mm)			
Pre-op	54.6 ± 2.6 (49.5–58.7)	46.8 ± 3.7 (43.6–53.5)	** < **0.001 (HS)
Post-op	46.6 ± 1.9 (44–51)	39.6 ± 4.8 (36–48.7)	0.015 (S)
Final follow-up	47.3 ± 2.5 (44.5–52)	47 ± 10 (36–58)	0.942 (NS)

Assessment of the outcome of the radiographic parameters of patients in the cannulated screw group and the K-wire group is shown separately in another table (Table 4).

**Table 4:** Radiographic outcome measurement of the included patients in the two study groups

Item	Measurements			*P*-values		
	Pre-op	Post-op	Final follow-up	Pre-op vs post-op	Pre-op vs final	Post-op vs final
Cannulated screw group						
Angle of Gissane	130.2 ± 9.8	129.04 ± 4.12	127.9 ± 6.7	0.333	0.221	0.28
Böhler’s angle	9.7 ± 11.6	25.2 ± 7.2	24.3 ± 7.7	0.000026	0.000074	0.365
Posterior facet inclination angle	48.3 ± 10.6	58.3 ± 9.1	58.03 ± 9.2	0.003	0.0036	0.471
Calcaneal height	44.8 ± 3.04	49.7 ± 1.9	49.3 ± 2	< 0.00001	< 0.00001	0.252
Calcaneal length	85.05 ± 4.6	88.6 ± 3. 9	88.5 ± 3.4	0.01	0.008	0.489
Calcaneal width	54.6 ± 2.6	46.6 ± 1.9	47.3 ± 2.5	< 0.00001	0.167	0.167
K-wire group						
Angle of Gissane	125.85 ± 16.34	122.17 ± 7.46	111.89 ± 13.82	0.415	0.010	0.009
Böhler’s angle	9.95 ± 11.27	27.24 ± 6.73	22.2 ± 7.11	0.001*	0.003*	0.034
Posterior facet inclination angle	44.34 ± 14.29	57.07 ± 14.1	51.23 ± 12.99	0.008	0.004	0.241
Calcaneal height	34.10 ± 3.09	42.27 ± 5.47	41.44 ± 4.61	0.004	0.007	0.007
Calcaneal length	69.77 ± 11.15	76.22 ± 12.78	78.03 ± 10.85	0.009	0.008	0.280
Calcaneal width	46.77 ± 3.70	39.63 ± 4.76	47.0 ± 10.15	0.002	0.623	0.030

^*^Wilcoxon test was used instead of paired *t*-test due to non-parametric data

### Factors affecting the outcomes

The factors associated with good outcomes were correction of final Böhler’s angle and final calcaneal length in the K-wire group, and correction of final posterior facet inclination angle in the cannulated screw group. The only factor associated with worse functional outcomes in both groups was development of subtalar arthritis (Table 5).

**Table 5:** Factors affecting functional outcomes according to AOFAS score in the two study groups

		Cannulated screw group				K-wire group			
		AOFAS score			*P*-value	AOFAS score			*P*-value
		Excellent	Good	Fair		Excellent	Good	Fair	
Age (years)^a^		35.5 ± 10.9	32 ± 8.6	37.5 ± 3.5	0.77^c^	54 ± 1.4	31.2 ± 8.5	36.2 ± 12	0.051^c^
Smoking^b^	Yes	3 (37.5%)	2 (25%)	3 (37.5%)	0.10^d^	0	4 (80%)	1 (20%)	0.19^d^
	No	7 (77.8%)	2 (22.2%)	0		2 (16.7%)	4 (33.3%)	6 (50%)	
Sex^b^	Male	9 (56.3%)	4 (25.0%)	3 (18.8%)	0.69^d^	1 (9%)	5 (45.5%)	5 (45.5%)	0.84^d^
	Female	1 (100%)	0	0		1 (16.7%)	3 (50%)	2 (33.3%)	
Side of injury^b^	Right	4 (50%)	3 (37.5%)	1 (12.5%)	0.456^d^	1 (16.7%)	2 (33.3%)	3 (50%)	0.871^d^
	Left	3 (100%)	0	0		1 (20%)	2 (40%)	2 (40%)	
	Bilateral	1 (33.3%)	1 (33.3%)	1 (33.3%)		0	2 (66.7%)	1 (33.3%)	
Time lapsed from trauma to surgery (days)^a^		4.13 ± 3.2	10.25 ± 5.56	5 ± 1.414	0.071^c^	7	6.67 ± 5.785	2.83 ± 2.317	0.267^c^
Sanders classification^b^	Type II	3 (60%)	1 (20%)	1 (20%)	0.097^d^	1 (20%)	3 (60%)	1 (20%)	0.49^d^
	Type III	7 (58.3%)	3 (25%)	2 (16.7%)		1 (8.3%)	5 (41.7%)	6 (50%)	
Essex-Lopresti classification^b^	Joint-depression	7 (58.3%)	3 (25%)	2 (16.7%)	0.97^d^	2 (15.4%)	6 (46.1%)	5 (38.5%)	0.696^d^
	Tongue-type	3 (60%)	1 (20%)	1 (20%)		0	2 (50%)	2 (50%)	
Subtalar OA (PGS)^b^	Grade 0	8 (72.7)	3 (27.3)	0	0.001^d^	2 (25%)	6 (75%)	0	0.027^d^
	Grade 1	2 (50%)	1 (25%)	1 (25%)		0	2 (28.6%)	5 (71.4%)	
	Grade 2	0	0	2 (100%)		0	0	2 (100%)	
Final calcaneal angle measurements (°)^a^	Böhler’s angle	25.6 ± 6.9	24.2 ± 5.5	20 ± 13.2	0.57^c^	32.7 ± 8.5	22.8 ± 6.2	18.2 ± 3.6	0.024^c^
	Gissane angle	126.4 ± 3.9	128.1 ± 5.8	132.7 ± 14.2	0.40^c^	109.8 ± 24.2	117.0 ± 4.0	108.3 ± 16.9	0.611^c^
	Posterior facet inclination angle	62.4 ± 7.1	54.0 ± 2.3	48.9 ± 13.9	0.04^c^	68.8 ± 18.4	46.7 ± 12.4	49.2 ± 8.1	0.1^c^
Final calcaneal distance measurements (mm)^a^	Height	49.7 ± 1.9	48.8 ± 2.7	48.6 ± 0.8	0.61^c^	35.0	41.8 ± 4.8	43.5 ± 2.1	0.345^c^
	Length	88.2 ± 3.4	87.6 ± 4.3	91.0 ± 1.0	0.39^c^	71.0	80.2 ± 4.1	85	0.01^c^
	Width	46.6 ± 2.3	47.6 ± 1.1	49.3 ± 3.8	0.23^c^	36.0	46.3 ± 11.4	54.0 ± 2.8	0.412^c^
Mechanism of injury^b^	Falling from height	9 (60%)	4 (26.7%)	2 (13.3%)	0.23^d^	2 (15.4%)	4 (30.8%)	7 (53.8%)	0.21^d^
	Falling down stairs	0	0	1 (100%)		0	1 (100%)	0	
	Road traffic accidents	1 (100%)	0	0		0	3 (100%)	0	
Associated injuries^b^	Yes	0	1 (33.3%)	2 (66.7%)	0.396^d^	0	3 (37.5%)	5 (62.5%)	0.204^d^
	No	10 (71.4%)	3 (21.4%)	1 (7.2%)		2 (22.2%)	5 (55.6%)	2 (22.2%)	
Number of screws or K-wires^b^	2	3 (50%)	1 (16.7%)	2 (33.3%)	0.369^d^	0	1 (100%)	0	0.196^d^
	3	5 (62.5%)	3 (37.5%)	0		0	1 (50%)	1 (50%)	
	4	2 (66.7%)	0	1 (33.3%)		0	3 (75%)	1 (25%)	
	5	0	0	0		1 (11.1%)	3 (33.3%)	5 (55.6%)	
	6	0	0	0		1 (100%)	0	0	
Subtalar ROM (°)^a^		24.5 ± 5.0	23.8 ± 2.5	18.3 ± 5.8	0.17^c^	25	17.5 ± 4.6	17.9 ± 3.9	0.098^c^

^a^Data are presented as mean ± standard deviation

^b^Data are presented as no. (%)

^c^One-way ANOVA

^d^Chi-square test

## Discussion

The most important finding of our study, after a mean follow-up period of 12.4 months in the cannulated screw group, and 12.14 months in the K-wire group, was that the mean AOFAS score at the final follow-up was significantly higher among the cannulated screw group at 85.88 ± 8.34 (range, 70–100) points compared to that among the K-wire group at 75.82 ± 9.73 (range, 60 − 90) points (*P*-value = 0.0014). Satisfactory (excellent and good) outcomes between two groups were higher among the cannulated screw group (82.35%) compared to those among the K-wire group (58.82%) (*P*-value = 0.016), which signified that the cannulated screw technique had more curative impact than the K-wire technique.

Multiple scores for evaluation of the functional outcomes of treatment of DIACFs had been used by different authors: AOFAS score [3, 17], Maryland Foot Score [18], and Creighton–Nebraska Health Foundation Assessment score [4, 19]. We had used the AOFAS score in this study because it provides the best comparison tool between different studies [13]. The AOFAS score survey includes a mixture of questions that were both subjective and objective in nature.

The mean AOFAS score in the cannulated screw group of the current study was better than that of Tomesen et al. [20], who reported a mean AOFAS score 84.1 after treating 39 DIACFs using cannulated screws, with a mean follow-up of 66 months. Tomesen et al. [20] reported a post-operative wound infection of 13% compared to no infection in this group of the current study. Abdelazeem et al. [21] treated 33 patients with unilateral DIACFs Sanders type II and III with a limited open sinus tarsi approach and screw fixation; they reported satisfactory outcomes with a mean AOFAS score of 91.73 after a mean follow-up period of 28.8 months. Gavlik et al. [5] treated 15 patients with only Sanders type II fractures by arthroscopic-assisted fixation with three to six cancellous screws; their mean AOFAS was 93.7.

The mean AOFAS score in the K-wire group of this study was comparable to that in the study of Ebraheim et al. [22], who retrospectively reviewed 99 patients with 106 DIACFs managed by a limited sinus tarsi approach and trans-articular fixation with one or several pins, and showed that the mean AOFAS score was 77.6 (range, 31–91) at an average follow-up of 29 months. In Arora et al. [23], who treated 23 DIACFs in 19 patients with K-wire fixation, their mean AOFAS score was 85.1 (75 to 94), with excellent and good results that were obtained in 68.4% of patients at 26-month follow-up.

Comparing these current results with other types of MIS techniques in DIACFs, the satisfactory results in our study were comparable to those achieved by Cao et al. [24], who treated 33 patients with DIACFs with minimally invasive calcaneal locked plate, and their mean AOFAS score was 82. El-Mowafi et al. [6] reviewed 40 patients with 48 DIACFs who were treated with the Ilizarov technique and reported that the mean AOFAS score was 84.6. Also, the results in this current study were comparable to those achieved by Peng et al. [25], who treated 21 feet with DIACFs by augmentation of cannulated screws with injectable calcium phosphate, and their mean AOFAS was 84. The current results were lower than those achieved by Rammelt et al. [26]; they performed arthroscopically assisted screw fixation in 61 patients with Sanders type II DIACFs with a mean AOFAS score of 92.1.

Comparing these current results with ORIF in DIACFs, the satisfactory results in our study were higher than those achieved by Santosha et al. [27], who treated 30 DIACFs in 24 patients by ORIF and locking calcaneal plate and found that the mean AOFAS score was 79.9 at 24-month follow-up. The results in this current study were matched with those achieved by Wang et al. [28], who treated 47 patients (50 feet) with DIACFs by calcaneal plates; after a follow-up of 12–34 months, the rate of excellent and good results was 80%, with the mean AOFAS score of 86.8. The higher excellent and good results in our study as compared with ORIF were attributed to MIS techniques without extensive soft tissue dissection.

In the current study, pain was assessed with VAS for pain. In the cannulated screw group, the mean VAS decreased from 6.8 ± 0.9 pre-operatively to 3.2 ± 1.3 at the fourth week post-operatively, and to one ± 0.83 at final follow-up. In the K-wire group, the mean VAS decreased from 7.3 ± 0.5 pre-operatively to 3.5 ± 0.9 at the fourth week post-operatively, and to 1.7 ± 0.85 at final follow-up. The mean VAS showed non-significant differences between the two groups both pre-operatively and the fourth week post-operatively, but the final VAS was significantly better among the cannulated screw group compared to the K-wire group. Chen et al. [29] treated 20 patients with Sanders type III DIACFs; their mean post-operative VAS was 1.6 ± 1.35. Long et al. [30] retrospectively reviewed 32 patients with 33 DIACFs treated by percutaneous screw fixation, and found that the average VAS was 3.1 ± 1.6 at final follow-up.

Achievement of normal ROM of ankle and subtalar joints of the affected foot is considered one of the most important goals of treatment of DIACFs. At final follow-up, all patients of the current study showed normal ROM of the ankle joint, with no ankle arthritis noted. The normal ROM of the subtalar joint is 25°–30° in inversion and 5°–10° in eversion, and it was reported to have great variations in the literature 20°–60°, with inversion greater than eversion [31]. In the current study, the mean subtalar ROM (inversion and eversion) was significantly higher among the cannulated screw group (25 ± 5.3 (range, 15–35)) compared to the K-wire group (21.2 ± 5.2 (range, 15–30) degrees) (*P*-value = 0.02). In the cannulated screw group, the subtalar ROM ≥ 25° was achieved in 13 feet (76.5%), and in four feet (23.5%) the subtalar ROM was < 25°. In the K-wire group, the subtalar ROM ≥ 25° was achieved in 7 feet (50%), and in 7 feet (50%), the subtalar ROM was < 25°. Schepers et al. [32] reported that the average ROM of subtalar joint was 20° (range, 5°–40°), 67% of the normal value, and the average sagittal ROM of ankle joint was 53° (range 25°–75°), which represents 88% of the normal ROM. Comparing these current results with ORIF, similar mobility in the ankle and subtalar joints has been reported after ORIF with a plate [33, 34]. Grün et al. [35] followed up 25 patients with 26 DIACFs (Sanders II and III), treated with percutaneous and arthroscopically assisted calcaneal osteosynthesis with 12-month follow-up, and they found that the median ROM of ankle joint was 40° (33–90), representing 72% of the ROM of the uninjured joint, and the median ROM of the subtalar joint was 22° (5–37), representing 73% of the ROM of the uninjured joint. Walde et al. [36] reported 34 (50.7%) of their patients had a restricted ROM up to 15° and more than half (58.2%) of the patients had achieved more than 75% of their total ROM in the lower ankle joint.

In the current study, the mean full weight-bearing time was significantly longer among the K-wire group at 14.4 ± 1.8 (range, 10–16) weeks compared to that among the cannulated screw group at 12 ± 1 (range, 10–14) weeks (*P*-value = 0.0001). The mean time needed for patients to return to work was significantly longer among the K-wire group at 5.4 ± 1 (range, 3–7) months compared to that among the cannulated screw group at 4.3 ± 0.8 (range, 3–6) months (*P*-value = 0.003). Swords et al. [37] reviewed DIACFs that were treated with MIS calcaneal locked plate and found that 85% of patients were able to return to work at 16.5 weeks (range, 9–22 weeks). Kumar et al. [19] compared ORIF with MIS in managing DIACFs and reported that the average time to return to work in MIS was 14 weeks and that in ORIF was 16 weeks.

In the current study, the screws used ranged two to four screws; two were needed in six (35.3%) feet, three in eight (47%) feet, and four in three (17.7) feet. The K-wires used ranged two to six K-wires; two were needed in one (5.9%) foot, three in two (11.7%) feet, four in four (23.5%) feet, five in nine (53%) feet, and six in one (5.9%) foot. Walde et al. [36] reported four to seven K-wires were used. Gavlik et al. [5] used three to six cancellous screws.

Radiographic evaluation in the current study included measurement of three calcaneal angles which are; angle of Gissane, Böhler’s angle, and posterior facet inclination angle, and three calcaneal distances (mm) which are the height, length, and width of the calcaneus.

Regarding the mean values of three calcaneal angles, no statistically significant differences were found between the two groups, either pre-operatively, post-operatively, or at the final follow-up except for both of the post-operative and final follow-up measurements of the angle of Gissane which were lower among the K-wire group compared to that among the cannulated screw group, with statistically significant differences (Table 3). Regarding the mean values of the three calcaneal distances, the pre-operative, post-operative, and final follow-up measurements of the calcaneal height, length, and width were lower among the K-wire group compared to those among the cannulated screw group, with statistically significant differences except for both post-operative calcaneal length and final follow-up calcaneal width which showed no statistically significant differences between two groups (Table 3).

Assessment of radiographic outcomes of patients in the cannulated screw group showed significant improvement of post-operative and final follow-up measurements of all radiographic parameters compared to pre-operative measurements with exception of measurements of angle of Gissane and pre-operative versus final follow-up measurement of calcaneal width. There was a statistically non-significant change in post-operative versus final follow-up measurements of all radiographic parameters which signified that there was maintenance of correction that was obtained post-operatively (Table 4). Abdelazeem et al. [21] followed their patients radiographically by Böhler’s angle only and reported improvement in Böhler’s angle in all patients. The mean pre-operative angle was 2.8° (range from − 38° to 24°), and post-operatively it was 19.4° (range from 5° to 49°), but they did not give a correlation between radiographic correction and functional outcomes. Kapil Mani et al. [38], who used MIS with cannulated screws for treatment of DIACFs, reported the pre-operative calcaneal length, height, width, Böhler’s angle, and Gissane angle were significantly improved after surgery, but they did not give a correlation between radiographic correction and functional outcomes.

Assessment of radiographic outcomes of patients in the K-wire group showed significant improvement of post-operative and final follow-up measurements of all radiographic parameters compared to pre-operative measurements with exception of the measurements of the following: pre-operative versus post-operative measurements of the angle of Gissane, post-operative versus final follow-up posterior facet inclination angle, post-operative versus final follow-up calcaneal length, and pre-operative versus post-operative calcaneal width (Table 4). Arora et al. [23] reported that mean pre-operative Böhler’s angle was 3.04°, the post-operative mean angle was 18.9°, and the mean final follow-up measurement was 15.6°. Respective values for the Gissane angle were 113.52°, 129.1°, and 128.6°. There was a significant change in the Böhler’s angle immediately post-operative compared with pre-operative values (*P* = 0.035). Walde et al. [36] reported that Böhler’s angle was restored in 70.1% (47 of 67) of cases.

In the current study, the mean time of radiographic solid union was 8.9 ± 1.6 weeks in the cannulated screw group and 10.1 ± 1.5 weeks in the K-wire group. Longer duration was needed for union in the K-wire group (*P*-value = 0.016). Kapil Mani et al. [38] obtained union at 11.06 ± 1.82 (range, 8–16) weeks. Arora et al. [23] obtained union at a mean of 8.2 weeks.

In our study, subtalar arthritis was evaluated by PGS as it is reliable and easy for everyday clinical purposes [16]. In the cannulated screw group, subtalar joint arthritis of grade 1 was encountered in four (23.5%) feet, grade 2 in two (11.8%) feet, and there were no cases of grade 3. In the K-wire group, subtalar joint arthritis of grade 1 was encountered in seven (41.2%) feet, grade 2 in two (11.8%) feet, and there were no cases of grade 3 arthritis. Schepers et al. [32] showed subtalar arthritis in 49.3% of patients. El-Mowafi et al. [6] reported 43.8% of patients had subtalar arthritis. All patients with subtalar arthritis in our study were of mild to moderate degrees with no severe cases and were managed conservatively, and none of them needed subtalar fusion until the last follow-up.

Regarding other complications in our study, there were no severe complications as wound infection, non-union, or osteomyelitis; however, minor complications had occurred. In the K-wire group, 3 feet (17.65%) had superficial pin track infections which were treated by dressings and antibiotics, with no deep infections reported in both groups. In the cannulated screw group, one foot (5.9%) developed peroneal subluxation and tendinitis which were managed conservatively, and one foot (5.9%) developed prominent screw which was removed after union. Dewall et al. [39] retrospectively reported significantly lower wound complications in the percutaneous reduction group (6%) compared with the ORIF group (35.3%) and emphasized on the need for prospective study of percutaneous technique. Kumar et al. [19] compared ORIF with MIS in managing DIACFs in a prospective study, and reported seven of 23 fractures (30.4%) had wound problems in the ORIF group, and none in the MIS group (*P* = 0.005).

We did not use bone grafts after the reduction as the calcaneus is a spongy bone and has the ability for bone formation. Buckley [40] mentioned that the use of bone graft in treatment of DIACFs was non-mandatory.

MIS techniques of fixation of DIACFs with cannulated screws or K-wires had the advantages of early surgical intervention, shorter hospital stay, and avoidance of wound complications associated with ORIF (e.g., wound dehiscence, infection, and necrosis). Drilling a hole over the guide wires at the screw entry only by a cannulated drill bit had ensured good purchase for screws in the cancellous bone. K-wires had the advantage of easy removal as an outpatient procedure.

Our study had some limitations, including the relatively low number of patients and short follow-up period; however, it emphasized on the efficacy and the efficiency of MIS in treatment of DIACFs and achieving excellent functional and radiographic outcomes with low infection rates.

## Conclusion

Both techniques avoided wound complications associated with ORIF, with shorter hospital stay.

Patients in the cannulated screw group had better functional and radiographic outcomes, and a lower rate of subtalar arthritis than patients in the K-wire group. Cannulated screws had the ability of maintenance of correction that was obtained post-operatively more than K-wires.

Using K-wires had the advantage of decreased operative time than cannulated screws, and easy removal as an outpatient procedure.

Proper selection of patients, proper pre-operative planning, and strict careful application of both techniques are important for obtaining better functional outcomes and avoiding complications. A steep learning curve is required for both techniques.

Further studies on MIS techniques of fixation of DIACFs should be conducted with a higher number of patients and longer follow-up period.
